# Supplementary figures and images for: Genome-Wide Identification of the Hypericum perforatum WRKY Gene Family Implicates HpWRKY85 in Drought Resistance
Source: Int J Mol Sci. 2022 Dec 26;24(1):352. doi: 10.3390/ijms24010352 (PMC9820127; doi:10.3390/ijms24010352)

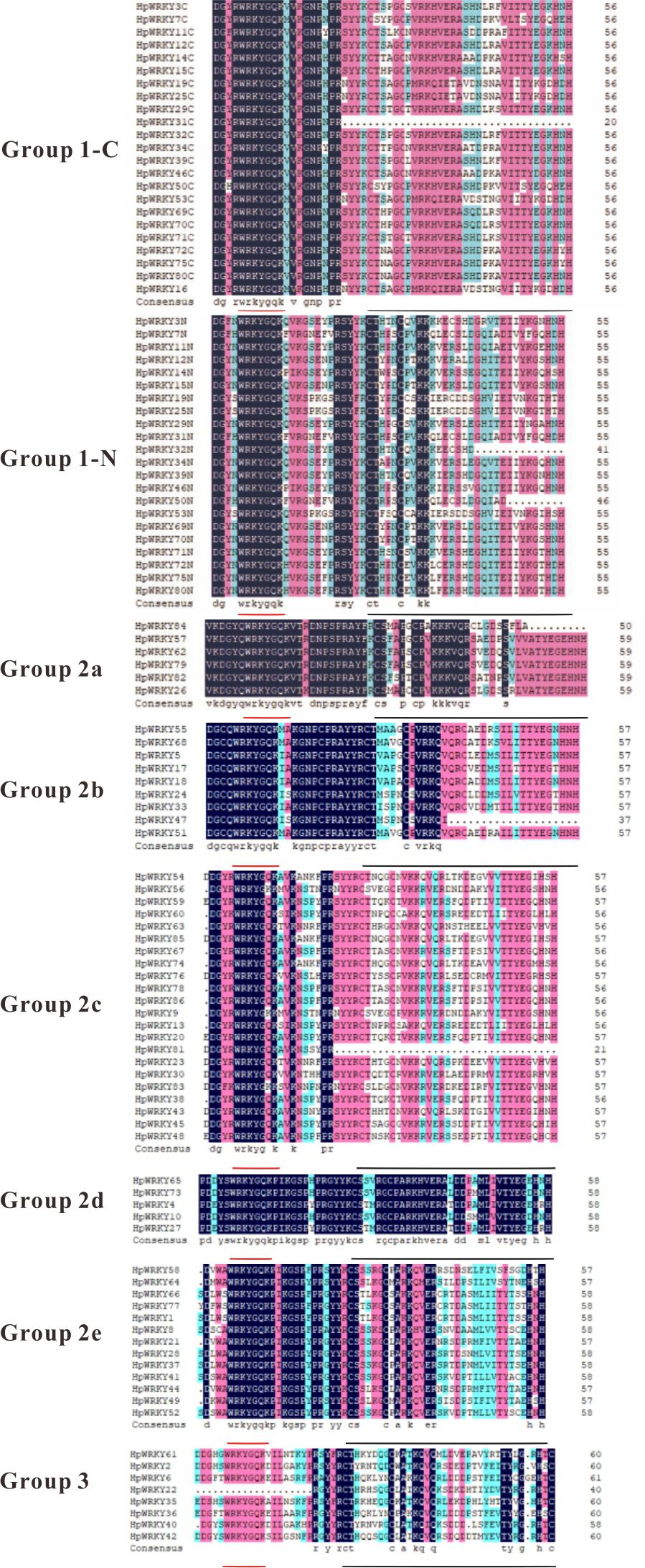

Supplement: Supplementary file 1 [file ijms-24-00352-s001.zip › Figure S1.jpg]

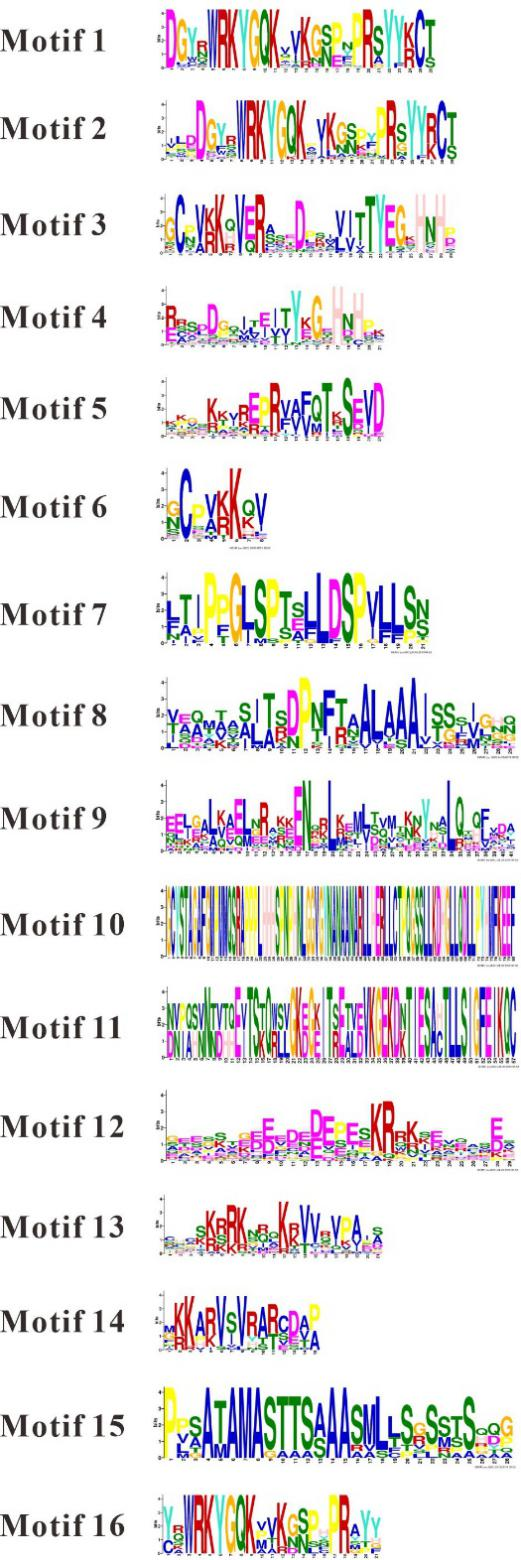

Supplement: Supplementary file 1 [file ijms-24-00352-s001.zip › Figure S2.png]

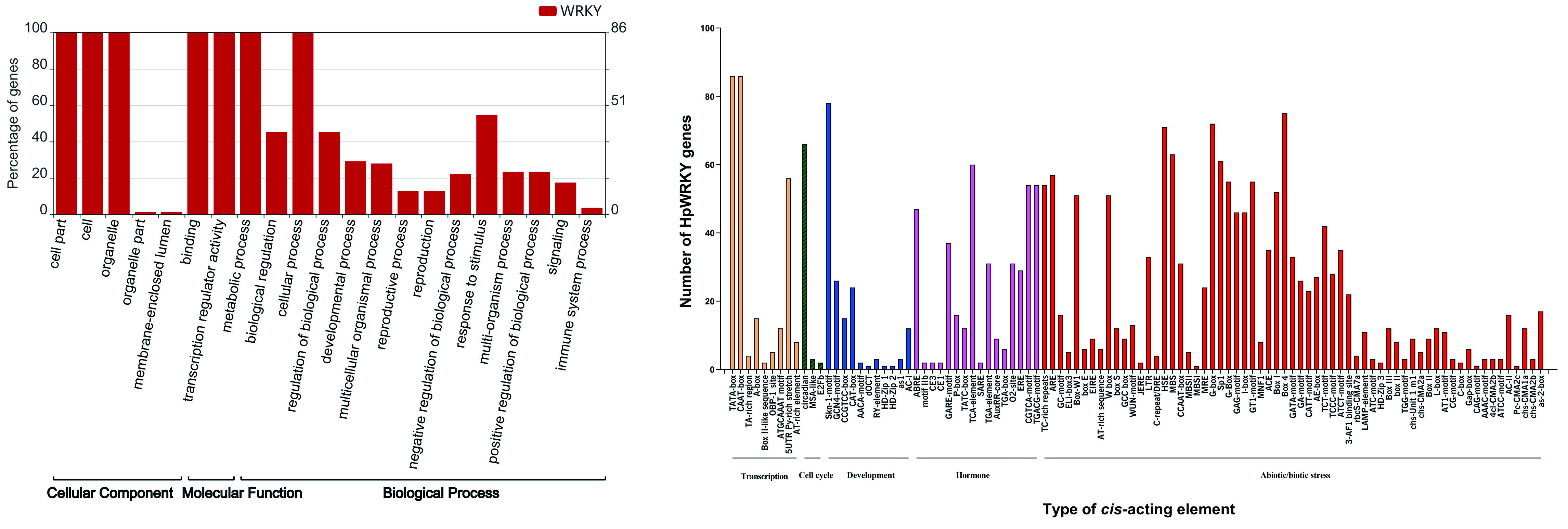

Supplement: Supplementary file 1 [file ijms-24-00352-s001.zip › Figure S3.jpg]

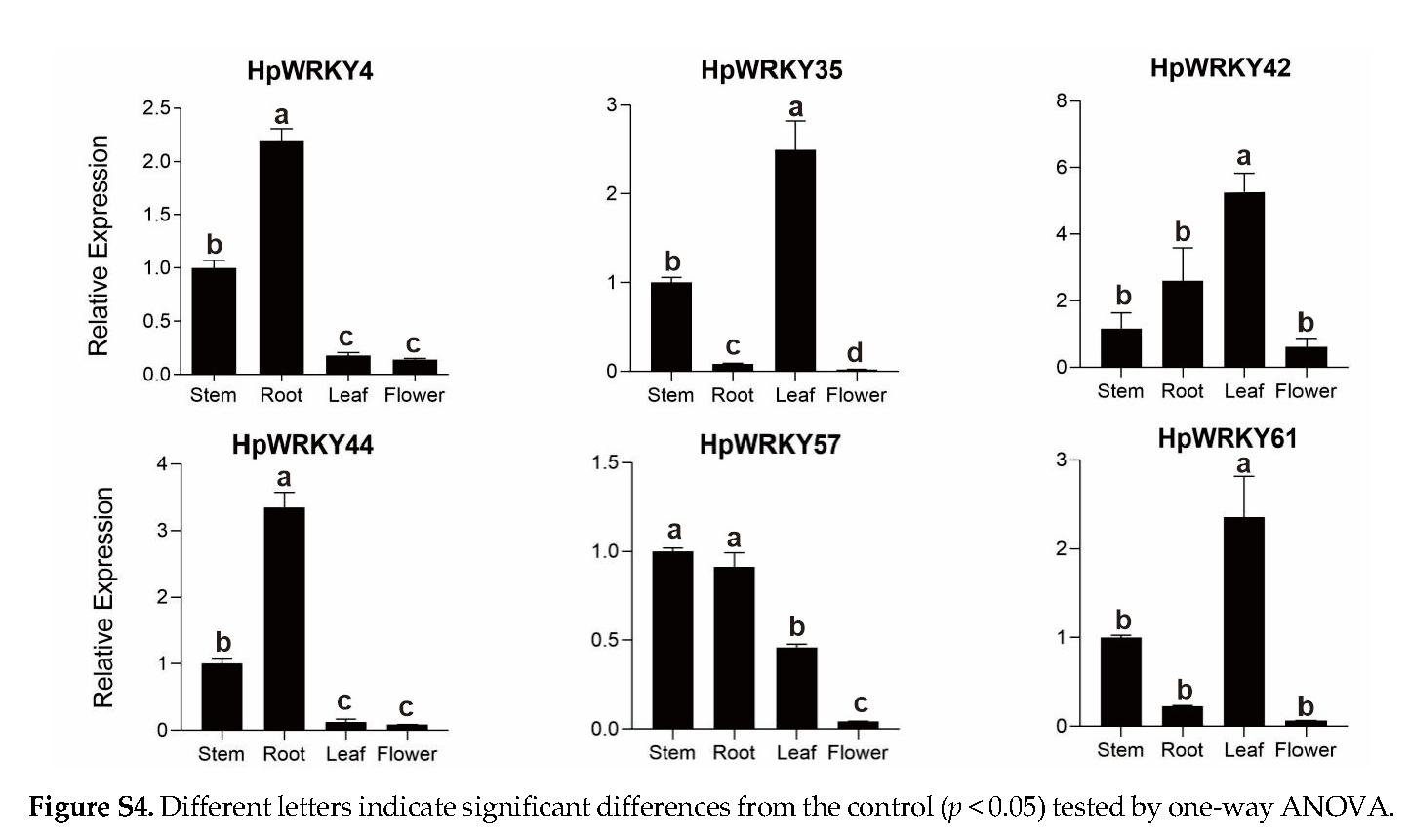

Supplement: Supplementary file 1 [file ijms-24-00352-s001.zip › Figure S4.png]
